# Acyl‐CoA synthetase long chain family member 4 plays detrimental role in early brain injury after subarachnoid hemorrhage in rats by inducing ferroptosis

**DOI:** 10.1111/cns.13548

**Published:** 2020-12-12

**Authors:** Xiao‐feng Qu, Tian‐yu Liang, De‐gang Wu, Nian‐sheng Lai, Ru‐ming Deng, Chao Ma, Xiang Li, Hai‐ying Li, Yi‐zhi Liu, Hai‐tao Shen, Gang Chen

**Affiliations:** ^1^ Department of Neurosurgery & Brain and Nerve Research Laboratory The First Affiliated Hospital of Soochow University Suzhou, Jiangsu Province China; ^2^ Department of Neurology The First People’s Hospital of Yancheng Yancheng, Jiangsu Province China; ^3^ Department of Neurosurgery The First Affiliated Hospital of Wannan Medical College Wuhu, Anhui Province China; ^4^ Department of Neurosurgery The People’s Hospital of Bozhou Bozhou, Anhui Province China

**Keywords:** acyl‐CoA synthetase long chain family member 4, early brain injury, ferroptosis, subarachnoid hemorrhage

## Abstract

**Aims:**

Acyl‐CoA synthetase long chain family member 4 (ACSL4) is closely related to tumor genesis and development in certain tissues. However, the function of ACSL4 in early brain injury (EBI) caused by subarachnoid hemorrhage (SAH) is unclear. In this study, we investigated the expression patterns and role of ACSL4 in SAH and post‐SAH EBI using a rat model of SAH.

**Methods:**

The rat model of SAH was induced by autologous blood injection into the prechiasmatic cistern of rats. We also used two specific inhibitors of ferroptosis (Ferrostatin‐1 and Liproxstatin‐1) to investigate the role of ferroptosis in EBI.

**Results:**

We found that ACSL4 levels in brain tissue increased significantly in post‐SAH EBI. Inhibiting the expression of ACSL4 using small interfering RNAs alleviated inflammation, blood‐brain barrier (BBB) impairment, oxidative stress, brain edema, and behavioral and cognitive deficits, and increased the number of surviving neurons, after SAH. Similar effects were obtained by suppressing ferroptosis.

**Conclusions:**

ACSL4 exacerbated SAH‐induced EBI by mediating ferroptosis. These findings may provide a theoretical basis for potential therapy aimed at alleviating post‐SAH EBI.

## Graphical abstract

ACSL4 levels in brain tissue of rats increase significantly in EBI and brain damage after SAH could be reduced by down‐regulation of ACSL4. ACSL4 could trigger ferroptosis and aggravate brain damage via catalyzing lipid metabolism. It may provide a theoretical basis for potential therapy to alleviate EBI after SAH

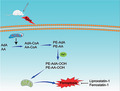

## 1 INTRODUCTION

Subarachnoid hemorrhage (SAH) is a cerebrovascular disease that shows high rates of morbidity and mortality; the annual incidence of SAH is also relatively high.^1^, ^2^ SAH is most commonly caused by ruptured intracranial aneurysms.^3^ Current treatments of intracranial aneurysms include endovascular coiling and neurosurgical clipping. However, the repair of ruptured aneurysms does not fully cure individuals who have already had these aneurysms because some of the SAH‐induced neurological impairments are permanent in these patients. Patients that survive a ruptured aneurism often suffer from varying degrees of neurocognitive impairment, which can adversely impact work and quality of life.^4^ For these reasons, this patient population urgently needs interventions that ameliorate and even reverse SAH‐induced pathophysiologic events.

Early brain injury (EBI), which encompasses the entirety of brain injury occurring in the 72 h after SAH, is associated with oxidative stress, neuroinflammation, neuronal death, blood‐brain barrier (BBB) damage, and dysfunction of brain autoregulation.^5^ EBI is one of the main pathologic events responsible for poor prognosis in SAH patients.^6^, ^7^ Early intervention during EBI can reduce neurologic dysfunction after SAH.^8^, ^9^ Brain edema is the main pathologic cause of post‐SAH EBI.^10^ Thus, improving our understanding of the pathogenic events involved in EBI will help in the development of novel interventions that will improve the prognosis of patients with SAH.

Acyl‐CoA synthetase long chain family member 4 (ACSL4) is a member of the long chain acyl coenzyme A synthase family (ACSLs), which is a class of essential enzymes involved in fatty acid metabolism. ACSL4 is mainly distributed in steroid‐producing tissues, especially in the adrenal glands and ovaries. ACSL4 can catalyze the synthesis of acyl‐CoA from fatty acids, which is an essential step in the process of fatty acid metabolism.^11^ Interestingly, ACSL4 serves as a key enzyme in the process of ferroptosis,^12^ which is a recently discovered form of oxidative cell death induced by small‐molecule substances.^13^ Unlike necrosis, ferroptosis is a regulated process of cell death caused by an imbalanced production and degradation of reactive oxygen species (ROS) within cells. The morphological characteristics of ferroptosis include a complete and non‐ruptured cell membrane, concentration of mitochondria and thickening of the mitochondrial membrane, and intact nucleus with no chromosomal condensation. Increased expression of ACSL4 promotes ferroptosis, and both promote the progression of several neurodegenerative diseases.^14^, ^15^, ^16^, ^17^

Post‐SAH EBI is associated with oxidative stress, but few studies have examined the role of ACSL4 in SAH‐induced EBI. We hypothesized that ROS‐related ferroptosis and ACSL4 may be critically involved in the process of EBI. Hence, in our present study, we investigated the activity of ACSL4 in a rat model of SAH. Because a previous study has convincingly demonstrated that ACSL4 is a key enzyme in the process of ferroptosis,^12^ we also investigated whether ferroptosis is involved in EBI, and examined ACSL4‐related mechanisms in a rat model of SAH using inhibitors of ferroptosis. The findings obtained in our present study will provide a theoretical basis for the identification of potential therapeutic targets, leading to the development of new therapies for patients with post‐SAH EBI.

## 2 MATERIALS AND METHODS

### 2.1 Animals

Adult male Sprague‐Dawley (SD) rats (300‐350 g) were purchased from the Experimental Animal Center of Shanghai (Shanghai, China). All procedures involving animals were approved by the Animal Care and Use Committee of the First Affiliated Hospital of Soochow University and were performed in accordance with the guidelines of the National Institutes of Health (Bethesda, MA, USA). The rats received food and water ad libitum and were housed under a constant temperature of 22‐24℃ and 40% humidity with a 12‐h light/dark cycle.

### 2.2 Establishment of the SAH model in *vivo*

SAH was induced by injecting autologous blood into the prechiasmatic cistern of rats as described in our previous study.^18^ Autologous blood was extracted from each rat’ s femoral artery using the microinjector. Briefly, the rats were anesthetized with an intraperitoneal injection of 4% chloral hydrate (10 ml/kg) and were then positioned on a stereotaxic frame. Next, a side‐port needle (having a rounded tip and an opening on the side), with its opening facing the right side, was inserted at a 45° angle to the sagittal plane, 7.5 mm anterior to bregma at the midline (location of the prechiasmatic cistern). The tip of the needle was positioned 2‐3 mm anterior to the chiasma and then retracted 0.5 mm. Bone wax was used to prevent blood and cerebrospinal fluid (CSF) leakage when necessary. Next, 0.3 ml fresh non‐heparinized blood, collected from the rat femoral artery, was slowly injected into the prechiasmatic cistern for 20 s using a microinjector. Rats in the Sham group were injected with 0.3 ml saline solution. To prevent dehydration, the rats were injected with 5 ml 0.9% saline after the induction of SAH. After a 45‐min recovery, all the rats were returned to their cages. The typical samples of SAH brain tissue contained blood at the bottom of the temporal lobe. Figure S1A shows an example of SAH brain tissue around the temporal lobe.

### 2.3 Experimental design

For Experiment 1, 42 rats (6 rats from the Sham group and 36 rats that survived the induction of SAH) were randomly divided into 7 groups (with 6 rats per group) as follows: 1 Sham group and 6 experimental groups; the rats were euthanized at the following timepoints: 3, 6, 12, 24, 48, and 72 h after the induction of SAH. Briefly, at each indicated timepoint after SAH induction, the rats in the appropriate experimental group were deeply anesthetized by 4% chloral hydrate (10 ml/kg). For Western blotting, three rats per group were killed and then transcardially perfused with chilled PBS and the brain tissues were collected from the base of the temporal lobe and stored immediately −80℃. For immunofluorescence labeling, the other three rats in each group were killed and then transcardially perfused with chilled PBS. Coronal brain sections, containing the basal temporal lobe, were collected from rats in each group, fixed in 4% paraformaldehyde, and used for immunofluorescence labeling (Figure S1B).

For Experiment 2, 72 rats (18 rats from the Sham group and 54 rats that survived SAH induction) were divided randomly into 4 groups (with 18 rats per group) as follows: Sham group, SAH group, SAH + negative control siRNA‐treated (Si‐control) group, and SAH + ACSL4 SiRNA‐treated (Si‐ACSL4) group (Figure S1C). At 24 h before SAH induction, rats in the SAH + Si‐control group and SAH + Si‐ACSL4 group were treated with the appropriate siRNA via injection into the brain ventricle. New rats were randomly selected to supplement the number of rats in each group when any of the experimental rats died prematurely after SAH induction.

For Experiment 3, we used 5 groups (18 rats from the Sham group and 72 rats that survived SAH induction, with 18 rats per group) as follows: Sham group, SAH group, SAH + Vehicle group, SAH + Ferrostatin‐1 group, and SAH + Liproxstatin‐1 group (Figure S1D). At 2 h before SAH induction, the rats in the Vehicle, Ferrostatin‐1, and Liproxstatin‐1 groups were intraperitoneally injected with their respective treatment. New rats were randomly selected to supplement the number of rats in each group when any of the rats died prematurely after SAH induction.

For Experiments 2 and 3, 18 rats in each group were randomly divided into 3 subgroups (with 6 rats per subgroup) using a table of random numbers by a researcher who did not participate in our present study. Entire coronal sections containing temporal base brain tissues were extracted from three rats in subgroup 1 and used for immunofluorescence and Nissl labeling. The underlying temporal base brain tissues of the other three rats were collected and frozen −80℃ for subsequent Western blotting, BBB permeability assay, and ROS assay. All the rats in subgroups 2 and 3 were examined for behavioral impairment at 24 h after SAH induction. Next, blood and CSF samples were collected from six rats in subgroup 2, and these rats were then sacrificed for detection of brain edema. The six rats in subgroup 3 were used for the Morris Water Maze text. Assessment of behavioral impairment, Morris Water Maze test, and detection of brain edema were performed by researchers blinded to the experimental groups. In quantitative analyses using Western blotting, BBB permeability assay, and ROS assay, each “n” represents individual data collected from one independent experiment using one rat, while combined data were obtained from at least six independent experiments using three rats per group. For immunofluorescence analyses and Nissl staining, representative images from at least six independent experiments using three rats per group are shown.

### 2.4 Drug administration

The specific inhibitors of ferroptosis used in this study were Ferrostatin‐1^19^ and Liproxstatin‐1^20^ (both obtained from Selleckchem). According to the manufacturer's instructions, Ferrostatin‐1 and Liproxstatin‐1 were dissolved in dimethyl sulfoxide (DMSO) and diluted with 0.9% normal saline to final the concentrations of 5 mg/ml and 3 mg/ml, respectively. These two inhibitors were then administered to the rats at the doses of 2 mg/kg and 5 mg/kg, respectively, via intraperitoneal injection at 2 h before the induction of SAH.

### 2.5 Transfection of small interfering RNA (siRNA)

To silence ACSL4 transcription, we used siRNA targeted to rat ACSL4 mRNA (Si‐ACSL4) as described previously.^18^ Additionally, we used scramble siRNA as a control treatment (Si‐control; both siRNAs were obtained from GenScript, Nanjing, China). According to the manufacturer's instructions, 500 pmol scramble siRNA and 500 pmol ACSL4 siRNA were separately dissolved in 5 μl RNase‐free water. Then, 10 μl Entranster‐in vivo RNA transfection reagent (Engreen) was added to each solution and mixed for 15 min. The resultant siRNA mixture was injected into the rat brain ventricle at 24 h before SAH induction. The ACSL4 siRNA sequences were as follows: (sense) 5’‐GCTCTGTCACGCACTTCGA dTdT‐3’ and (antisense) 3’‐dTdT UCGAAGUGCGUGACAGAGC‐5’.

### 2.6 Western blotting

Western blotting was performed as described previously.^21^ Brain tissues were obtained from the base of the temporal lobe and stored immediately at −80℃ until further use. For Western blotting, these brain samples were dissected with a chisel and mechanically disassociated in lysis buffer and phenylmethanesulfonyl fluoride (PMSF, both from Beyotime, China) mixed at a volume ratio of 100:1. Protein concentrations were determined using bicinchoninic acid (BCA) assay (Beyotime, China). Equivalent amounts of protein samples from each group (30μg/lane) were loaded onto 10% SDS‐polyacrylamide gels and then electrotransferred onto polyvinylidene difluoride membranes (PVDF, Merck Millipore). The membranes were blocked with 5% evaporated milk at room temperature (25℃) to prevent nonspecific binding. These membranes were incubated with the primary antibodies anti‐ACSL4 (Santa Cruz Biotechnology), antialbumin (Abcam), and anti‐β‐tubulin (Santa Cruz Biotechnology) at 4℃ overnight, and then with a horseradish peroxidase (HRP)‐linked secondary antibody (Santa Cruz Biotechnology) at room temperature for 1 h. Protein signals were detected using an enhanced chemiluminescence (ECL) kit (Thermo Fisher Scientific). The relative densities of the bands were analyzed using ImageJ Software (NIH), and data were normalized to the expression level of β‐tubulin, used as loading control.

### 2.7 Immunofluorescence labeling

Coronal brain sections containing the basal temporal lobe were analyzed using immunofluorescence labeling as described previously.^22^ After collection, the brain tissues were fixed in 4% paraformaldehyde, subjected to standard histologic processing, and embedded in paraffin after equally cut into 4μm. The paraffin‐embedded tissues were sectioned, dewaxed, and blocked in 5% bovine serum to prevent nonspecific binding. These sections were then incubated with primary antibodies specific for ACSL4 (Abcam) and NeuN (Abcam) at 4℃ at least 8 h. After subsequent incubation with Alexa Fluor 488 donkey anti‐mouse IgG and Alexa Fluor 555 donkey anti‐rabbit IgG (Life Technologies), these sections were treated with antifading mounting medium containing 4, 6‐diamidino‐2‐phenylindole (DAPI, Southern Biotech) and coverslipped. Relative fluorescence intensity was analyzed using ImageJ software (NIH).

### 2.8 ELISA

Blood and CSF samples, collected from the rats, were centrifuged, and supernatants were processed for ELISA. Briefly, after the blood samples were collected using the femoral artery, samples were stored in serum‐separating tubes for 2 h at room temperature, and then centrifuged at 2000 rpm at 4℃ for 20 min to separate the serum. The obtained sera were stored at −80℃. CSF samples were obtained from the rat foramen magnum and then stored at −80℃. Concentrations IL‐1β in the blood and CSF samples were detected using the IL‐1β ELISA kit (Bio‐Swamp) having the sensitivity of ≥ 30 pg/ml. The kit was specifically used to detect IL‐1β and showed no obvious cross‐reactivity with other similar substances. Concentrations of TNF‐α in the blood and CSF samples were detected using a TNF‐α ELISA kit (Bio‐Swamp) having the sensitivity of ≤ 1.6 pg/ml. This kit was specifically used to detect TNF‐α and showed no obvious cross‐reactivity with other similar substances. These kits were used in accordance with the manufacturer's instructions.

### 2.9 Detection of reactive oxygen species (ROS)

We used the Reactive Oxygen Species Assay Kit (Beyotime) to detect the relative levels of ROS in brain tissue samples. The brain tissue samples were ground and centrifuged, and the supernatants from each sample were processed in accordance with the manufacturer's instructions. Each supernatant was mixed with the probe, incubated for 30 min at 37℃, and then washed using washing buffer in the kit. Fluorescence intensity of each group of samples was detected using a fluorometric microplate reader (FilterMax F5, Molecular Devices), and all data were normalized to those of the Sham group.

### 2.10 Blood‐brain barrier (BBB) injury

The level of albumin in brain tissue is very low because of the presence of the BBB. If the BBB is damaged, the content of albumin in brain tissue will increase significantly. Therefore, to evaluate the degree of BBB damage, we used Western blotting to measure the level of albumin in the brain tissue of rats from each group. Western blotting was conducted the same as described above, but in this procedure, the primary antibody was antialbumin (Abcam). The relative density of albumin bands was analyzed using ImageJ software, and expression levels were normalized to those of β‐tubulin, used as loading control.

### 2.11 Nissl staining

Nissl staining was used to visualize neuronal loss or survival in the hippocampus and temporal cortex as described previously.^22^ Surviving neurons with pale nuclei and large cellular bodies were counted and averaged. Neurons showing dark staining, and those with shrunken cellular bodies, were considered dead and excluded from counting. After sections of brain tissue were deparaffinized, toluidine blue solution was used to stain the sections for 50 min at 50‐60℃, after which the sections were subjected to sequential dehydration in 70%, 80%, 95%, 100% (I), and 100% ethanol (II), and then cleared in xylene (I) and xylene (II) for 5 min. Neutral resin was applied to the sections, and the sections were coverslipped. Nissl‐stained sections were evaluated under a light microscope (Nikon). Random fields in the temporal cortex near the blood clot and CA2 region of the hippocampus were selected and observed, and the numbers of surviving neurons were counted.

### 2.12 Brain edema

Rats were deeply anesthetized by 4% chloral hydrate (10 ml/kg) and sacrificed by decapitation; whole brains were rapidly harvested without perfusion. Blood from the brain surface was gently blotted with filter paper, and the brains were weighed immediately (wet weight). The brains were then baked at 100℃ for 72 h and weighed again to obtain the dry weight. The Elliot formula was used to calculate the brain water content as follows: brain water content (%) = (wet weight ‐ dry weight) / wet weight × 100%.

### 2.13 Behavioral examination

The 12 rats used in Experiments 2 and 3 were scored at 24 h after SAH induction using the following three criteria: appetite, activity, and neurological deficits. Scoring was performed by a researcher not participating in the study. Details on the scoring procedures are shown in Table 1.

**TABLE 1:** Neurobehavioral evaluation

Category	Behavior	Score
Appetite	Finished meal	0
	Left meal unfinished	1
	Scarcely ate	2
Activity	Walk and reach at least three corners of the cage	0
	Walk with some stimulations	1
	Almost always lying down	2
Deficits	No deficits	0
	Unstable walk	1
	Impossible to walk	2

### 2.14 The Morris water maze test

Learning abilities and memory of the rats after the induction of SAH were measured by the Morris water maze as described previously.^23^ In brief, the water maze (divided into four quadrants) had a total diameter of 120 cm, while the circular target platform had a diameter of 10 cm and a height of 40 cm. The target platform was wrapped in black rope and placed in the middle of the third quadrant, with the platform height 2 cm below the water surface. Water temperature was maintained at 25 ± 2℃, and the water was dyed black with ink. During the training and testing phase, the reference viewpoint around the pool remained unchanged. During the training phase, rats were placed into the pool from all four quadrants and given 60 s to locate the target platform. If the rats could not find the target platform within 60 s, a rod was used to gently guide them to the platform. After reaching the target platform, the rats were allowed to remain there for 10 s to develop a spatial memory for the location of the platform and were then removed from the maze. The training was performed for 5 consecutive days, and SAH was induced on day 6. The time from leaving the starting point to reaching the target platform was recorded and defined as escape latency (EL). During the test phase, rats were placed in randomly selected quadrants of the pool and allowed to find the target platform. EL and swimming distance were recorded during the next 3 days of testing. Average EL and distance were used as indicators of learning ability and cognitive function.

### 2.15 Statistical analysis

All data in this study are expressed as mean ± SEM. All experimental data were analyzed by GraphPad Prism 7.0 software and SPSS 20.0 software. Data obtained using the Morris Water Maze test were analyzed using two‐way repeated ANOVA. Two‐sided unpaired Student's t*‐*test was used to analyze statistical differences between two groups. Prior to statistical analyses, the data sets for each group were tested for normality of distribution using the Kolmogorov‐Smirnov test. Results were considered statistically significant at *p* < 0.05.

## 3 RESULTS

### 3.1 Mortality

Our monitoring data show no rat dead in the Sham group, while total mortality rate in the SAH groups was 20.2% (41/203) because of modeling. The mortality rates of rats in the SAH groups used in Experiments 1, 2, and 3 were 20%, 21.7%, and 19.1%, respectively, and the specific number of mortalities of rats in each group was shown in Table 2. To summarize, no significant difference in mortality was observed between the experimental groups. No significant increase in mortality was observed in rats treated with Ferrostatin‐1 or Liproxstatin‐1 after SAH induction.

**TABLE 2:** Mortalities detail in each experimental group

Experiment 1		Experiment 2		Experiment 3	
Group	Mortality /Totality	Group	Mortality /Totality	Group	Mortality /Totality
Sham	0/6	Sham	0/18	Sham	0/18
SAH 3h	2/8	SAH	5/23	SAH	4/22
SAH 6h	1/7	SAH + Si‐control	6/24	SAH + Vehicle	4/22
SAH 12h	1/7	SAH + Si‐ACSL4	4/22	SAH + Ferrostain‐1	5/23
SAH 24h	2/8			SAH + Liproxstatin‐1	4/22
SAH 48h	1/7				
SAH 72h	2/8				

### 3.2 ACSL4 levels are elevated in brain tissue after SAH induction

**FIGURE 1:**
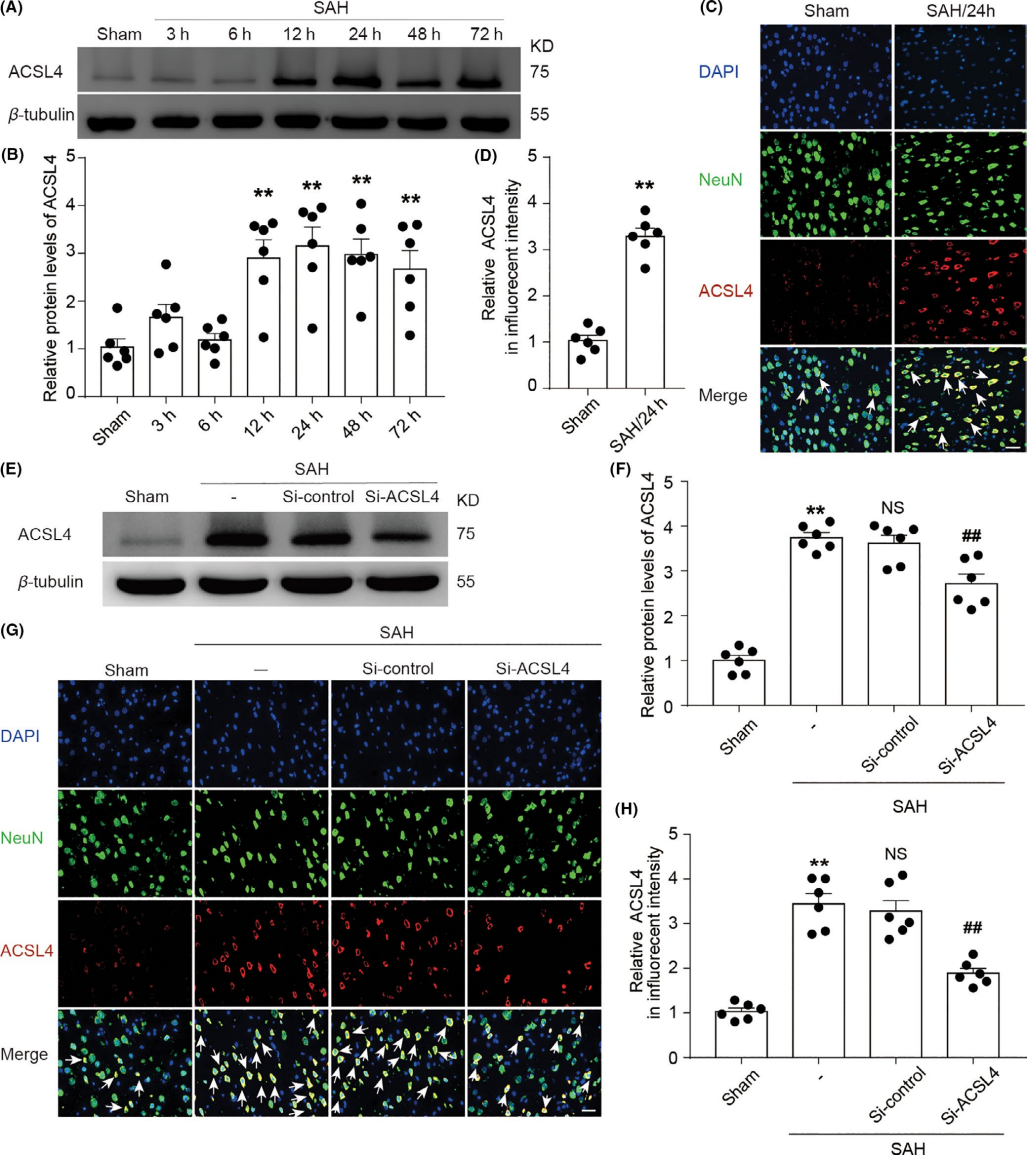
(A) Western blotting shows the levels of ACSL4 at 3, 6, 12, 24, 48, and 72 h after SAH induction. (B) Quantification analysis of relative ACSL4 levels at various time points. **p < 0.05 (*p* = 0.0012 in 12‐h group; *p* = 0.0006 in 24‐h group; *p* = 0.0004 in 48‐h group; *p* = 0.0033 in 72‐h group) vs. Sham group; results were analyzed using unpaired t*‐*test, *n *= 6. (C) Immunofluorescence analysis of brain sections using antibodies specific for ACSL4 (red), a neuronal marker (green), and DAPI nuclear stain (blue). Representative images of Sham and SAH 24‐h group are shown, and white arrows indicate ACSL4‐positive neurons. Scale bar = 100μm. (D) Quantification analysis of the relative fluorescence intensity of ACSL4 in Sham and SAH 24‐h group. **p = 0.0022 vs. Sham group; unpaired t‐test, *n *= 6. (E) Western blotting shows the levels of ACSL4 in Sham, SAH, SAH + Si‐control, and SAH + Si‐ACSL4 groups. (F) Quantification analysis of the relative levels of ACSL4 in various groups at 24 h after SAH induction. **p < 0.0001 vs. Sham group; ^##^ *p* = 0.0090 vs. SAH + Si‐control group; results analyzed using unpaired t‐test, *n *= 6. NS, no significant difference (*p* = 0.5761) vs. SAH group; unpaired t‐test, *n *= 6. (G) Immunofluorescence analysis of brain sections at 24 h after SAH induction using antibodies specific for ACSL4 (red), a neuronal marker (green), and DAPI nuclear stain (blue). Scale bar = 100μm. (H) Quantification analysis of the relative fluorescence intensity of ACSL4. **p < 0.0001 vs. Sham group; ^##^ *p* = 0.0004 vs. SAH + Si‐control group; results analyzed using unpaired t‐test, *n *= 6. NS, no significant difference (*p* = 0.6287) vs. SAH group; unpaired t‐test, *n *= 6. All data are presented as mean ± SEM. Mean values of the Sham group were normalized to 1.0

In order to determine the protein level of ACSL4 in brain tissue after SAH induction, we collected brain tissue samples at different timepoints after SAH induction and analyzed the expression of ACSL4 using Western blotting. Compared with that of the Sham group, the expression of ACSL4 began to increase at 12 h after SAH induction and reached its highest level at 24 h after the induction of SAH; this high level of ACSL4 lasted for 3 days (*p* < 0.05, *n *= 6; Figure 1A,B). We further evaluated the level of ACSL4 using immunofluorescence labeling. The level of ACSL4 in neurons was significantly increased at 24 h after SAH induction compared with that of the Sham group (*p* < 0.01, *n *= 6; Figure 1C,D), which was consistent with the result obtained using Western blotting. Therefore, 24 h after the induction of SAH was selected as the timepoint for the following two experiments conducted in this study.

### 3.3 Knockdown of ACSL4 ameliorates EBI after SAH

To study the role of ACSL4 in EBI after SAH induction in rats, we used transfection with Si‐ACSL4 to reduce the expression of ACSL4 (SAH + Si‐ACSL4 group); treatment with Si‐NC was used as a control treatment (SAH + Si‐control group). After the respective treatments, brain tissue was collected at 24 h after SAH induction for Western blotting and immunofluorescence labeling. Our results show that ACSL4 levels decreased markedly in the SAH + Si‐ACSL4 group compared with those of the SAH + Si‐control group (both *p* < 0.01, *n *= 6; Figure 1E–H).

**FIGURE 2:**
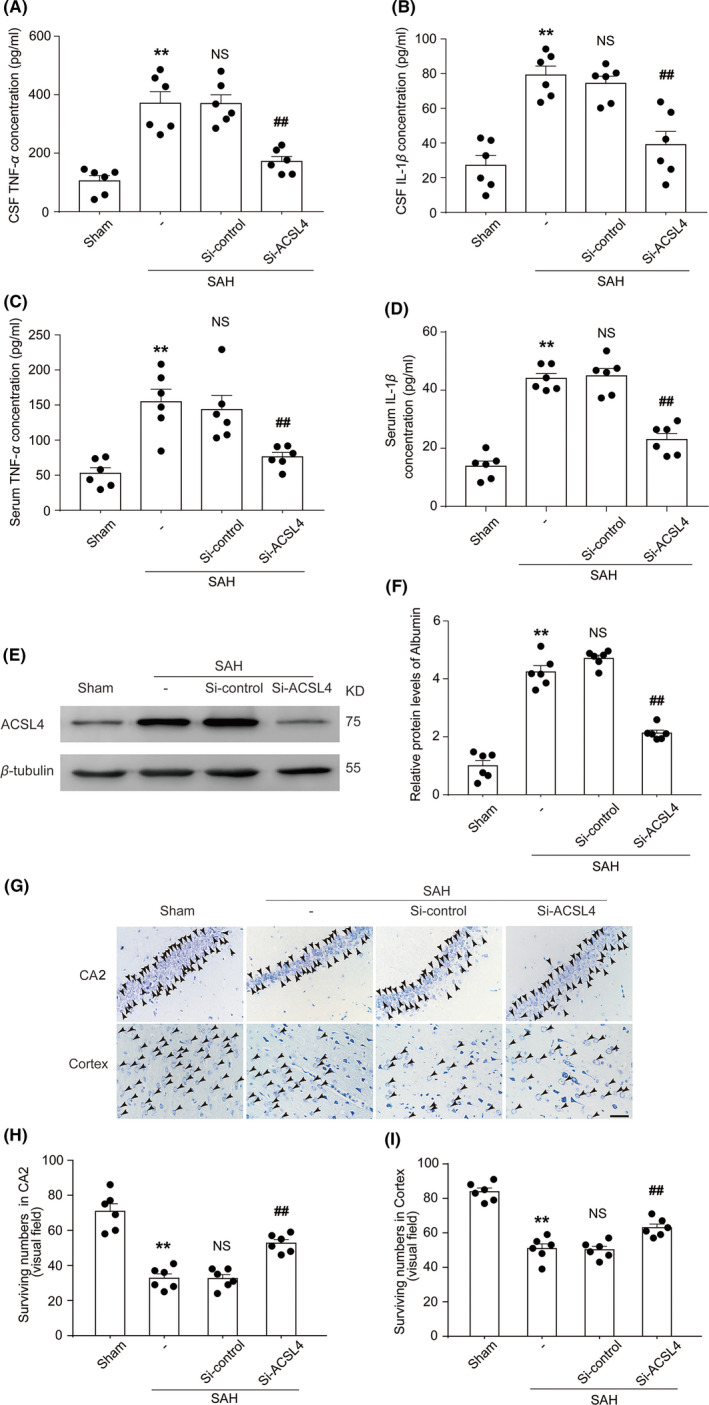
(A) Quantification analysis of TNF‐α concentrations in CSF samples obtained from rats in various groups at 24 h after SAH induction. **p = 0.0001 vs. Sham group; ^##^ *p* = 0.0002 vs. SAH + Si‐control group; results analyzed using unpaired t‐test, *n *= 6. NS, no significant difference (*p* = 0.9845) vs. SAH group; unpaired t‐test, *n *= 6. (B) Quantification analysis of IL‐1β concentration in CSF samples obtained from rats in various groups at 24 h after SAH induction. **p < 0.0001 vs. Sham group; ^##^ *p* = 0.0025 vs. SAH + Si‐control group; unpaired t‐test, *n *= 6. NS, no significant difference (*p* = 0.4889) vs. SAH group; results analyzed using unpaired t‐test, *n *= 6. (C) Quantification analysis of TNF‐α concentration in blood samples obtained from rats in various groups. **p = 0.0004 vs. Sham group; ^##^ *p* = 0.0094 vs. SAH + Si‐control group; results analyzed using unpaired t‐test, *n *= 6. NS, no significant difference (*p* = 0.6968) vs. SAH group; unpaired t‐test, *n *= 6. (D) Quantification analysis of IL‐1β concentration in blood samples obtained from rats in various groups. **p < 0.0001 vs. Sham group; ^##^ *p* < 0.0001 vs. SAH + Si‐control group; results analyzed using unpaired t‐test, *n *= 6. NS, no significant difference (*p* = 0.7763) vs. SAH group; unpaired t‐test, *n *= 6. (E) Western blotting shows albumin levels in brain tissue of rats in various groups at 24 h after SAH induction. (F) Quantification analysis of relative albumin levels in various groups. **p < 0.0001 vs. Sham group; ^##^ *p* < 0.0001 vs. SAH + Si‐control group; results analyzed using unpaired t‐test, *n *= 6. NS, no significant difference (*p* = 0.0852) vs. SAH group; unpaired t‐test, *n *= 6. (G) Nissl staining shows surviving neurons in the CA2 hippocampal region and temporal cortex of rats in various groups at 24 h after SAH induction black arrows indicate surviving neurons. Scale bar = 40µm. Quantification analysis of surviving neurons in the CA2 region of the hippocampus (H), **p < 0.0001 vs. Sham group; ^##^ *p* < 0.0001 vs. SAH + Si‐control group; results analyzed using unpaired t‐test, *n *= 6. NS, no significant difference (*p* = 0.9619) vs. SAH group; unpaired t‐test, *n *= 6. Quantification analysis of surviving neurons in the temporal cortex (I), **p < 0.0001 vs. Sham group; ^##^ *p* = 0.0014 vs. SAH + Si‐control group; results analyzed using unpaired t‐test, *n *= 6. NS, no significant difference (*p* = 0.8508) vs. SAH group; unpaired t‐test, *n *= 6. All data are shown as mean ± SEM. Mean values of the Sham group were normalized to 1.0

Next, we used ELISA to evaluate the effect of ACSL4 on the inflammatory response after SAH induction and to determine the levels of inflammatory cytokines (including those of IL‐1β and TNF‐α) in the sera and CSF samples collected from the rats in each group. Compared with those in the Sham group, concentrations of IL‐1β and TNF‐α in the SAH group were increased significantly. Additionally, the concentrations of IL‐1β and TNF‐α in the SAH + Si‐ACSL4 group were lower than those in the SAH + Si‐control group, indicating that ACSL4 exacerbated inflammation in rats after the induction of SAH (*p* < 0.05, *n *= 6; Figure 2A–D).

We then used Western blotting to measure the content of albumin in the brain tissue in order to evaluate the extent of injury to the BBB. Compared with those in the Sham and SAH + Si‐ACSL4 groups, respectively, the contents of albumin in the SAH and SAH + Si‐control group were significantly increased, which suggests that ACSL4 exacerbated SAH‐induced BBB injury in the rats (both *p* < 0.01, *n *= 6; Figure 2E,F). Nissl staining was used to observe the surviving neurons in rat brain tissues. Fewer surviving neurons were found in the cortex and the CA2 region of the hippocampus of the SAH group compared with those of the Sham group. The SAH + Si‐ACSL4 group had more surviving neurons than did the SAH + Si‐control group. These results suggest that reducing the expression of ACSL4 protected neurons after the induction of SAH (all *p* < 0.01, *n *= 6; Figure 2G–I).

**FIGURE 3:**
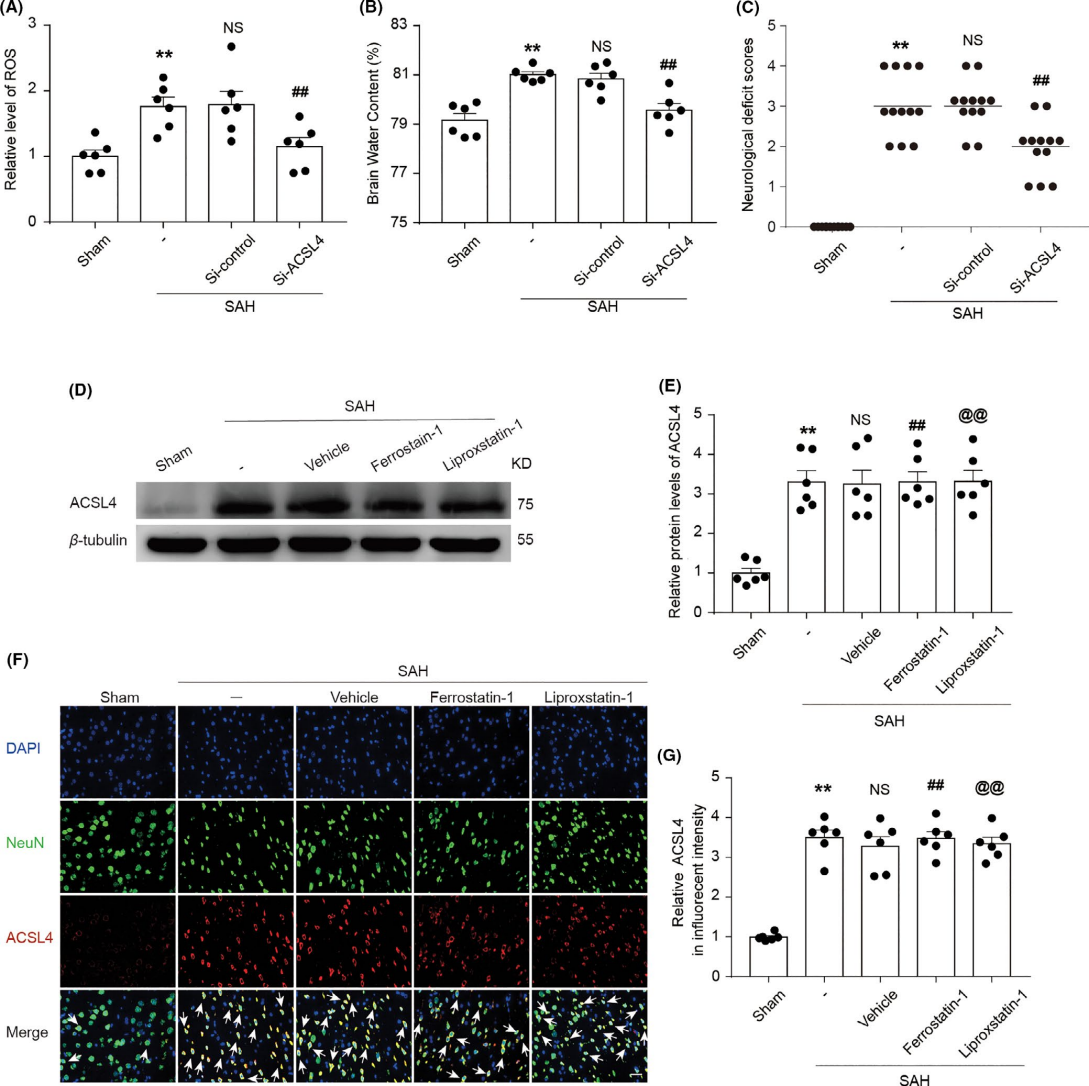
(A) Quantification analysis of relative ROS levels in brain tissue of rats in various groups at 24 h after SAH induction. (B) Graphs show brain water content of rats in various groups at 24 h after SAH induction. **p = 0.0012 vs. Sham group; ^##^ *p* = 0.0268 vs. SAH + Si‐control group; results analyzed using unpaired t‐test, *n *= 6. NS, no significant difference (*p* = 0.9135) vs. SAH group; unpaired t‐test, *n *= 6. (C) Neurological‐deficit scores of rats in various groups at 24 h after SAH induction, **p < 0.0001 vs. Sham group; ^##^ *p* = 0.0004 vs. SAH + Si‐control group; results analyzed using unpaired t‐test, *n *= 12. NS, no significant difference (*p* = 0.7747) vs. SAH group; unpaired t‐test, *n *= 12. (D) Western blotting shows ACSL4 levels in the Sham, SAH, SAH + Vehicle, SAH + Ferrostatin‐1, and SAH + Liproxstatin‐1 groups at 24 h after SAH induction. (E) Quantification analysis of relative ACSL4 levels in various groups. **p < 0.0001 vs. Sham group; ^##^ *p* = 0.8999, ^@@^ *p* = 0.8831 vs. SAH + Vehicle group; results analyzed using unpaired t‐test, *n *= 6. (F) Immunofluorescence analysis performed using antibodies specific for ACSL4 (red), a neuronal marker (green), and DAPI nuclear stain (blue). Scale bar = 100 μm. (G) Quantification analysis of the relative fluorescence intensity of ACSL4. **p < 0.0001 vs. Sham group; ^##^ *p* = 0.5136, ^@@^ *p* = 0.8177 vs. SAH + Vehicle group; results analyzed using unpaired t*‐*test, *n *= 6. All data are shown as mean ± SEM. Mean values of the Sham group were normalized to 1.0

ROS, which are single‐electron reduction products of oxygen, are indicative of oxidative stress. We found that ROS levels in the SAH group were significantly higher than those in the Sham group, while the ROS levels in the SAH + Si‐ACSL4 group were significantly lower than those in the SAH + Si‐control group. Collectively, these results suggested that reducing the expression of ACSL4 alleviated oxidative stress after SAH induction (both *p* < 0.05, *n *= 6; Figure 3A). Analysis of brain water content indicated that brain water content increased significantly in the SAH group compared with that of the Sham group. Compared with that of the SAH + Si‐control group, brain edema was decreased in the SAH + Si‐ACSL4 group. These results suggested that reducing the expression of ACSL4 alleviated the degree of brain edema after SAH induction (both *p* < 0.01, *n *= 6; Figure 3B). Neurological‐deficit scores of rats in the SAH and SAH + Si‐control groups were higher than those of rats in the Sham and SAH + Si‐ACSL4 group, respectively. This finding indicated that excessive ACSL4 expression exacerbated neurological deficits in rats after the induction of SAH (both *p* < 0.01, *n *= 12; Figure 3C).

### 3.4 Effect of Ferrostatin‐1 and Liproxstatin‐1 on EBI after SAH induction

**FIGURE 4:**
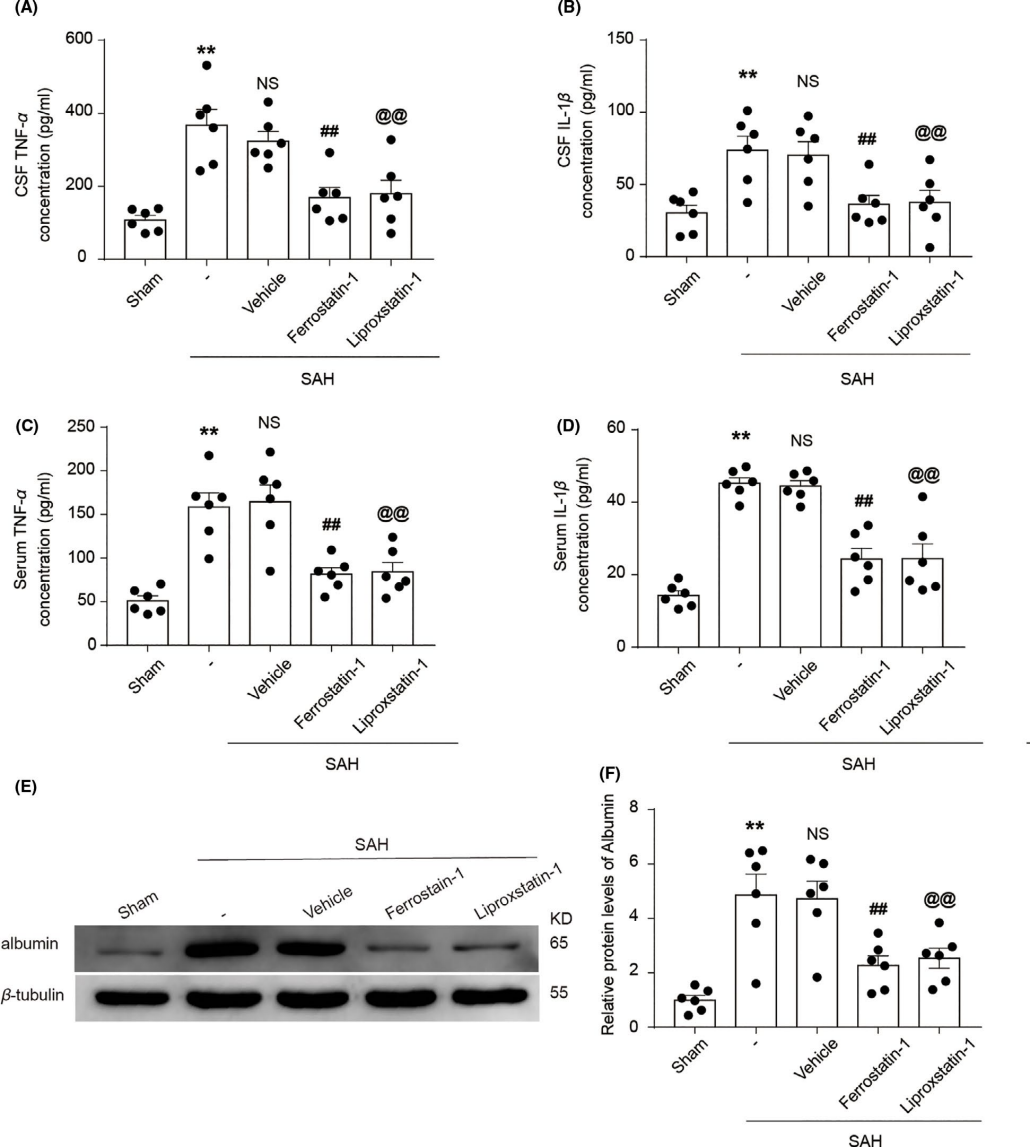
(A) Quantification analysis of TNF‐α concentration in CSF samples obtained from rats in various groups at 24 h after SAH induction. **p = 0.0002 vs. Sham group; ^##^ *p* = 0.0024, ^@@^ *p* = 0.0099 vs. SAH + Vehicle group; results analyzed using unpaired t*‐*test, *n *= 6. NS, no significant difference (*p* = 0.4169) vs. SAH group; unpaired t*‐*test, *n *= 6. (B) Quantification analysis of IL‐1β concentration in CSF samples obtained from rats in various groups. **p = 0.0030 vs. Sham group; ^##^ *p* = 0.0137, ^@@^ *p* = 0.0284 vs. SAH + Vehicle group; results analyzed using unpaired t*‐*test, *n *= 6. NS, no significant difference (*p* = 0.8027) vs. SAH group; unpaired t*‐*test, *n *= 6. (C) Quantification analysis of TNF‐α concentration in blood samples obtained from rats in various groups at 24 h after SAH induction. **p = 0.0001 vs. Sham group; ^##^ *p* = 0.0026, ^@@^ *p* = 0.0047 vs. SAH + Vehicle group; results analyzed using unpaired t*‐*test, *n *= 6. NS, no significant difference (*p* = 0.8128) vs. SAH group; unpaired t*‐*test, *n *= 6. (D) Quantification analysis of IL‐1β concentration in blood samples obtained from rats in various groups at 24 h after SAH induction. **p < 0.0001 vs. Sham group; ^##^ *p* = 0001, ^@@^ *p* = 0.0010 vs. SAH + Vehicle group; results analyzed using unpaired t*‐*test, *n *= 6. NS, no significant difference (*p* = 0.7443) vs. SAH group; unpaired t*‐*test, *n *= 6. (E) Western blotting shows albumin levels in brain tissue of rats in the Sham, SAH, SAH + Vehicle, SAH + Ferrostatin‐1, and SAH + Liproxstatin‐1 groups at 24 h after SAH induction. (F) Quantification analysis of relative albumin levels in various groups. **p = 0.0006 vs. Sham group; ^##^ *p* = 0.0076, ^@@^ *p* = 0.0147 vs. SAH + Vehicle group; results analyzed using unpaired t‐test, *n *= 6. NS, no significant difference (*p* = 0.8951) vs. SAH group; unpaired t‐test, *n *= 6. All data are shown as mean ± SEM. Mean values of the Sham group were normalized to 1.0

The results described above showed that ACSL4 aggravated EBI after SAH induction in rats, and previous studies have confirmed that ACSL4 can mediate ferroptosis. We hypothesized that ferroptosis is involved in EBI of rats after SAH induction. To verify this hypothesis, we used two specific inhibitors of ferroptosis (Ferrostatin‐1 and Liproxstatin‐1). The expression of ACSL4 in the SAH + Ferrostatin‐1 and SAH + Liproxstatin‐1 groups did not differ significantly from that in the SAH + Vehicle group (both *p* > 0.05, *n *= 6; Figure 3D–G). However, these inhibitors decreased the concentration of IL‐1β and TNF‐α in the sera and CSF samples obtained from rats after SAH induction (all *p* < 0.05, *n *= 6; Figure 4A–D). Compared with the levels of the rats in the SAH + Vehicle group, albumin levels in the brain tissue of rats in the SAH + Ferrostatin‐1 and SAH + Liproxstatin‐1 groups were decreased. This finding suggested that these two inhibitors alleviated BBB injury after SAH (both *p* < 0.05, *n *= 6; Figure 4E,F).

**FIGURE 5:**
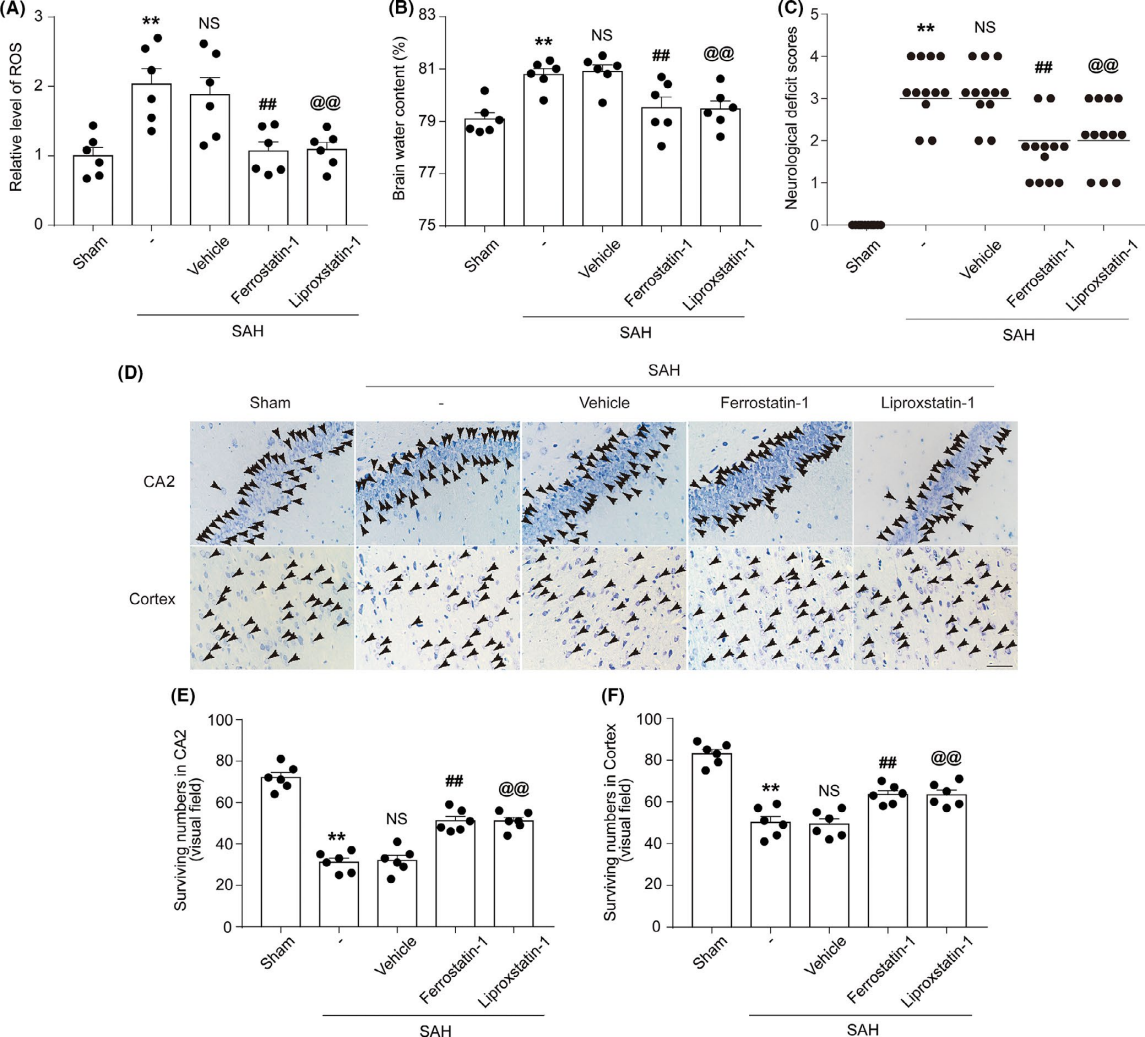
(A) Quantification analysis of relative ROS levels in brain tissue of rats in various groups at 24 h after SAH induction. **p = 0.0022 vs. Sham group; ^##^ *p* = 0.0165, ^@@^ *p* = 0.0150 vs. SAH + Vehicle group; results analyzed using unpaired t*‐*test, *n *= 6. NS, no significant difference (*p* = 0.6560) vs. SAH group; unpaired t*‐*test, *n *= 6. (B) Graphs show the brain water content of rats in various groups at 24 h after SAH induction. **p = 0.0004 vs. Sham group; ^##^ *p* = 0.0177, ^@@^ *p* = 0.0050 vs. SAH + Vehicle group; results analyzed using unpaired t‐test, *n *= 6. NS, no significant difference (*p* = 0.7463) vs. SAH group; unpaired t‐test, *n *= 6. (C) Neurological‐deficit scores of rats in various groups at 24 h after SAH induction. **p < 0.0001 vs. Sham group; ^##^ *p* = 0.0002, ^@@^ *p* = 0.0030 vs. SAH + Vehicle group; results analyzed using unpaired t*‐*test, *n *= 12. NS, no significant difference (*p* = 0.7713) vs. SAH group; unpaired t*‐*test, *n *= 12. (D) Nissl staining shows surviving neurons in the CA2 hippocampal region and temporal cortex of rats in various groups at 24 h after SAH induction; black arrows indicate surviving neurons. Scale bar = 40µm. (E) Quantification analysis of surviving neurons in the CA2 region of the hippocampus, **p < 0.0001 vs. Sham group; ^##^ *p* = 0.0002, ^@@^ *p* < 0.0001 vs. SAH + Vehicle group; results analyzed using unpaired t*‐*test, *n *= 6. NS, no significant difference (*p* = 0.7971) vs. SAH group; unpaired t*‐*test, *n *= 6. (F) Quantification analysis of surviving neurons in the temporal cortex, **p < 0.0001 vs. Sham group; ^##^ *p* = 0.0011, ^@@^ *p* = 0.0020 vs. SAH + Vehicle group; results analyzed using unpaired t*‐*test, *n *= 6. NS, no significant difference (*p* = 0.8322) vs. SAH group; unpaired t*‐*test, *n *= 6. All data are shown as mean ± SEM. Mean values of the Sham group were normalized to 1.0

ROS levels in the SAH + Ferrostatin‐1 and SAH + Liproxstatin‐1 group were lower than those of the rats in the SAH + Vehicle group (both *p* < 0.05, *n *= 6; Figure 5A). The degree of brain edema was also comparatively lower in the SAH + Ferrostatin‐1 and SAH + Liproxstatin‐1 group (both *p* < 0.05, *n *= 6; Figure 5B). Compared with those of rats in the SAH + Vehicle group, the rats in the SAH + Ferrostatin‐1 and SAH + Liproxstatin‐1 groups had lower neurological‐deficit scores (all *p* < 0.01, *n *= 12; Figure 5C) and more surviving neurons in the cortex and CA2 region of the hippocampus (all *p* < 0.01, *n *= 6; Figure 5D–F).

### 3.5 Treatment with Si‐ACSL4, Ferrostatin‐1, and Liproxstatin‐1 improves cognitive function after SAH induction

**FIGURE 6:**
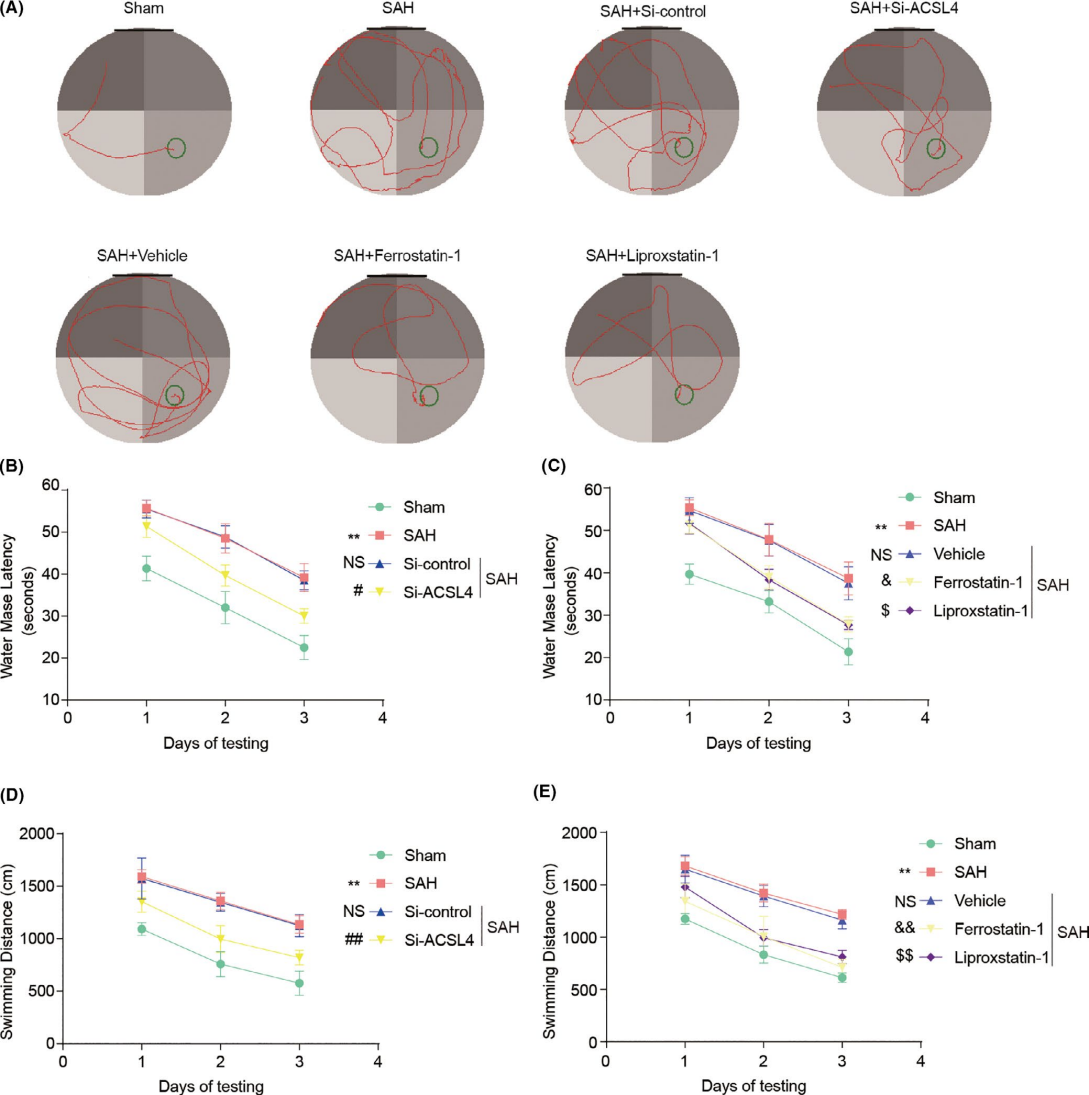
(A) Representative images of the Sham, SAH, SAH + Si‐control, SAH + Si‐ACSL4, SAH + Vehicle, SAH + Ferrostatin‐1, and SAH + Liproxstatin‐1 in the Morris water maze test. (B, C) Quantification analysis of the escape latency of rats in various groups. In B, **p < 0.0001 vs. Sham group; NS, no significant difference (*p* > 0.9999) vs. SAH group; ^#^ *p* = 0.0121 vs. SAH + Si‐control group; two‐way repeated‐measures ANOVA, row factor: groups, *p* < 0.0001; column factor: days, *p* < 0.0001; interactions: *p* = 0.9678. *n *= 6. In C, **p < 0.0001 vs. Sham group; NS, no significant difference (*p* > 0.9999) vs. SAH group; ^&^ *p* = 0.0281, ^$^ *p* = 0.0227 vs. SAH + Vehicle group; two‐way repeated‐measures ANOVA, row factor: groups, *p* < 0.0001; column factor: days, *p* < 0.0001; interactions: *p* = 0.8607. *n *= 6. (D, E) Quantification analysis of the swimming distance of rats in various groups. In D, **p < 0.0001 vs. Sham group; NS, no significant difference (*p* > 0.9999) vs. SAH group; ^##^ *p* = 0.0087 vs. SAH + Si‐control group; two‐way repeated‐measures ANOVA, row factor: groups, *p* < 0.0001; column factor: days, *p* < 0.0001; interactions: *p* = 0.9959. *n *= 6. In E, **p < 0.0001 vs. Sham group; NS, no significant difference (*p* > 0.9999) vs. SAH group; ^&&^ *p* = 0.0002, ^$$^ *p* = 0.004 vs. SAH + Vehicle group; two‐way repeated‐measures ANOVA, row factor: groups, *p* < 0.0001; column factor: days, *p* < 0.0001; interactions: *p* = 0.9648. *n *= 6. All data are shown as mean ± SEM

The learning ability and cognitive function of the rats were evaluated after the induction of SAH using the Morris water maze test. The results show that latency and swimming distance of rats in the SAH group were significantly higher than those of rats in the Sham group. After treatment with Si‐ACSL4, Ferrostatin‐1, and Liproxstatin‐1, the latency and swimming distance of rats in the SAH + Si‐ACSL4 group were significantly lower than those of rats in the SAH + Si‐control group. Similarly, the rats in the SAH + Ferrostatin‐1 and SAH + Liproxstatin‐1 groups exhibited significant improvements in these parameters compared with those of rats in the SAH + Vehicle group (all *p* < 0.05, *n *= 6; Figure 6).

## 4 DISCUSSION

Previous studies have suggested that pathophysiological changes from cerebral vasospasm cause poor prognosis in patients with SAH.^24^ However, recent studies have shown that adequate treatment of cerebral vasospasm does not effectively improve the prognosis of these patients.^25^ Several studies examining post‐SAH EBI have shown that EBI is an important factor affecting the prognosis of patients with SAH.^26^, ^27^ Therefore, improved understanding of the mechanisms driving EBI may provide novel therapies for improving the prognosis of patients with SAH.

Recent studies have shown that abnormal expression of ACSL4 in some tissues is related to tumor progression.^28^, ^29^ However, ACSL4 has not been studied in SAH‐induced EBI. To the best of our knowledge, our present study is the first to investigate the expression and role of ACSL4 in brain tissue after SAH. Western blotting analysis showed that the level of ACSL4 in rat brain tissue changed after SAH induction, with the levels of ACSL4 beginning to increase at 12 h after SAH induction and reaching their peak at 24 h after the induction of SAH; at 72 h post‐SAH, these levels were significantly higher than those of the rats in the Sham group. These results suggest that the level of ACSL4 increased in the time window of post‐SAH EBI; therefore, we hypothesized that ACSL4 may play a detrimental role in EBI. To verify the role of ACSL4 in EBI, we injected specific ACSL4 siRNA into the lateral ventricles of rats and found that knockdown of ACSL4 ameliorated the post‐SAH inflammatory response, BBB damage, oxidative stress, brain edema, and behavioral and cognitive impairment, and increased the number of surviving neurons. Our results indicate that ACSL4 expression was significantly increased in the brain tissue of rats after SAH induction and that EBI was partially alleviated by reducing the expression of ACSL4.

Ferroptosis is a form of iron‐dependent cell death caused by lethal lipid peroxidation.^13^ Ferroptosis‐associated lipid peroxidation, a harmful biological event caused by free radicals, can lead to pathological changes in cell membrane structure and functional damage of cellular components.^13^, ^19^, ^30^ Indeed, lipid peroxidation is an important component of ferroptosis and is involved in the pathological process of EBI. After SAH, red blood cells in the blood decompose in cerebrospinal fluid to release hemoglobin and then metabolize further, generating superoxide‐free radicals. The superoxide‐free radicals then produce lipid peroxides, leading to a series of brain‐damaging events.^31^, ^32^ Lipid peroxidation is mainly mediated by the content and localization of polyunsaturated fatty acids (PUFAs) with labile bis‐allylic hydrogen atoms, which can affect the normal execution of ferroptosis.^33^ PUFAs are involved in the synthesis of lipid signaling pathways, membrane phospholipid composition, and conduction of ferroptosis signaling after lipid oxidation, which induces ferroptosis in cells.^30^, ^34^ Phosphatidylethanol, containing arachidonic acid (AA) or adrenic acid (AdA), is an especially important lipid signaling molecule that can regulate lipid oxidation and drive cellular ferroptosis.^12^, ^30^ ACSL4 plays an important role in this process. As a lipid‐modulating enzyme, ACSL4 can activate long chain polyunsaturated fatty acids with a preference for AA and AdA, and can catalyze the synthesis of corresponding coenzymes,^35^ thereby participating in the synthesis of membrane phospholipids. Importantly, ACSL4 is essential in the induction of ferroptosis. A previous study, using mouse embryonic fibroblasts (MEF) to investigate ferroptosis, has shown that knockout of the *ACSL4* gene prevented the induction of ferroptosis. This finding indicates that ACSL4 can be used as an index to predict cellular initiation of ferroptosis.^12^ Specifically, ACSL4 participates in the synthesis of membrane phospholipids by activating PUFAs and can catalyze the synthesis of Acyl‐CoA from PUFAs in vivo as the first step of fatty acid catabolism. First, ACSL4 catalyzes the ligation of an AA or AdA to produce AA‐CoA or AdA‐CoA, respectively.^12^, ^34^ Then, Acyl‐CoA synthesizes phosphatidylethanolamine‐PUFA (mainly PE‐PUFA, PE‐AA, and PE‐AdA), which is catalyzed by lysophosphatidylcholine acyltransferase 3 (LPCAT3). Finally, the cytotoxic phosphatidylethanolamine hydroperoxides (mainly PE‐OOH, PE‐AA‐OOH, and PE‐AdA‐OOH) are synthesized with the participation of iron ions, ultimately leading to cellular ferroptosis^12^, ^33^, ^34^, ^36^ (Figure S2).

In previous studies, Park et al^37^ found that ACSL4 levels are high in K‐RAS mutant colorectal cancer cells, which are sensitive to ferroptosis. Additionally, bromelain induces an increase in ACSL4 expression and subsequent ferroptosis in these cells. Conversely, reducing the levels of ACSL4 using Si‐ACSL4 has been shown to reduce ferroptosis. Li et al^38^ used rosiglitazone and siRNA to reduce the expression of ACSL4 and found that low levels of ACSL4 can protect the body against ferroptosis and alleviate cellular damage and intestinal barrier dysfunction caused by intestinal ischemia/reperfusion injury. The results obtained in these studies indicate that ACSL4 participates in the pathologic processes of various diseases by regulating ferroptosis. However, whether ACSL4 participates in EBI by inducing ferroptosis remains unclear.

In our present study, we have shown that ACSL4 levels in the brain tissue of rats, which increased significantly in post‐SAH EBI and brain damage, could be reduced by downregulation of ACSL4 expression. Based on these results, we hypothesized that ACSL4 can trigger ferroptosis and aggravate brain damage by mediating lipid metabolism. We then used two specific inhibitors of ferroptosis (Ferrostatin‐1 and Liproxstatin‐1) to investigate whether ferroptosis is involved in the pathologic process of EBI. Our results show that in SAH‐induced rats, treatment with these inhibitors alleviated the neuroinflammation, BBB damage, oxidative stress, brain edema, and behavioral and cognitive impairments associated with post‐SAH EBI, and increased the number of surviving neurons. These results suggest that ferroptosis is indeed involved in EBI and that the degree of brain injury can be alleviated by reducing ferroptosis (Figure S2). However, the level of ACSL4 was not changed significantly by the administration of these inhibitors. We speculate that these inhibitors suppress ferroptosis not by inhibiting the expression of ACSL4 but rather by inhibiting the formation of enzymatic‐reaction products, or by directly inhibiting the occurrence of ferroptosis. This hypothesis, however, requires further investigation.

Our present study had several limitations. First, we used only young male rats, which prevented us from examining the effects of aging and estrogen on EBI. Also, the inhibitors of ferroptosis were administered prior to SAH induction, which affected the clinical relevance of this study. Moreover, these drugs were administered via intraperitoneal injection; therefore, their effects on the brain, especially on neuron integrity, may have been indirect. In future studies, we will further explore the role of ferroptosis in post‐SAH brain injury by administering the drugs after the induction of SAH; we will also explore the pharmacological and pharmacodynamic mechanisms of related drugs administered concurrently. Our study shows that ferroptosis likely occurs in post‐SAH injury. However, ferroptosis is not the only cell death pathway involved in post‐SAH EBI. Apoptosis and necroptosis also occur in brain tissues after SAH.^7^, ^18^ Additionally, several mechanisms, such as neuroinflammation, BBB damage, delayed cerebral ischemia, and blockage of cerebrospinal fluid flow, contribute to post‐SAH EBI.^1^ Ferroptosis, as a type of necrosis, can induce inflammation in tissues; therefore, it may contribute to neuroinflammation after the occurrence of SAH. In this study, treatment with Ferrostatin‐1 and Liproxstatin‐1 alleviated neuroinflammation in post‐SAH EBI. However, the relationships between ferroptosis and other cell death pathways in post‐SAH EBI remain unclear.

Presently, EBI is known to be related to necrosis, apoptosis, and other forms of cell death.^18^, ^39^, ^40^ In this study, we confirmed for the first time that EBI is also related to ferroptosis, and found that ACSL4 is an important modulator of ferroptosis. Our results show that brain injury was ameliorated not only by ferroptosis inhibitors but also by reducing the expression of ACSL4. Collectively, these findings provide novel therapeutic targets for the improved treatment of EBI.

## CONFLICT OF INTEREST

The authors declare no conflict of interest.

## Supporting information

Fig S1

Fig S2

## Data Availability

The data that support the findings of this study are available from the corresponding author upon reasonable request.
