# Supplementary figures and images for: Acyl‐CoA synthetase long chain family member 4 plays detrimental role in early brain injury after subarachnoid hemorrhage in rats by inducing ferroptosis
Source: CNS Neurosci Ther. 2020 Dec 12;27(4):449–63. doi: 10.1111/cns.13548 (PMC7941219; doi:10.1111/cns.13548)

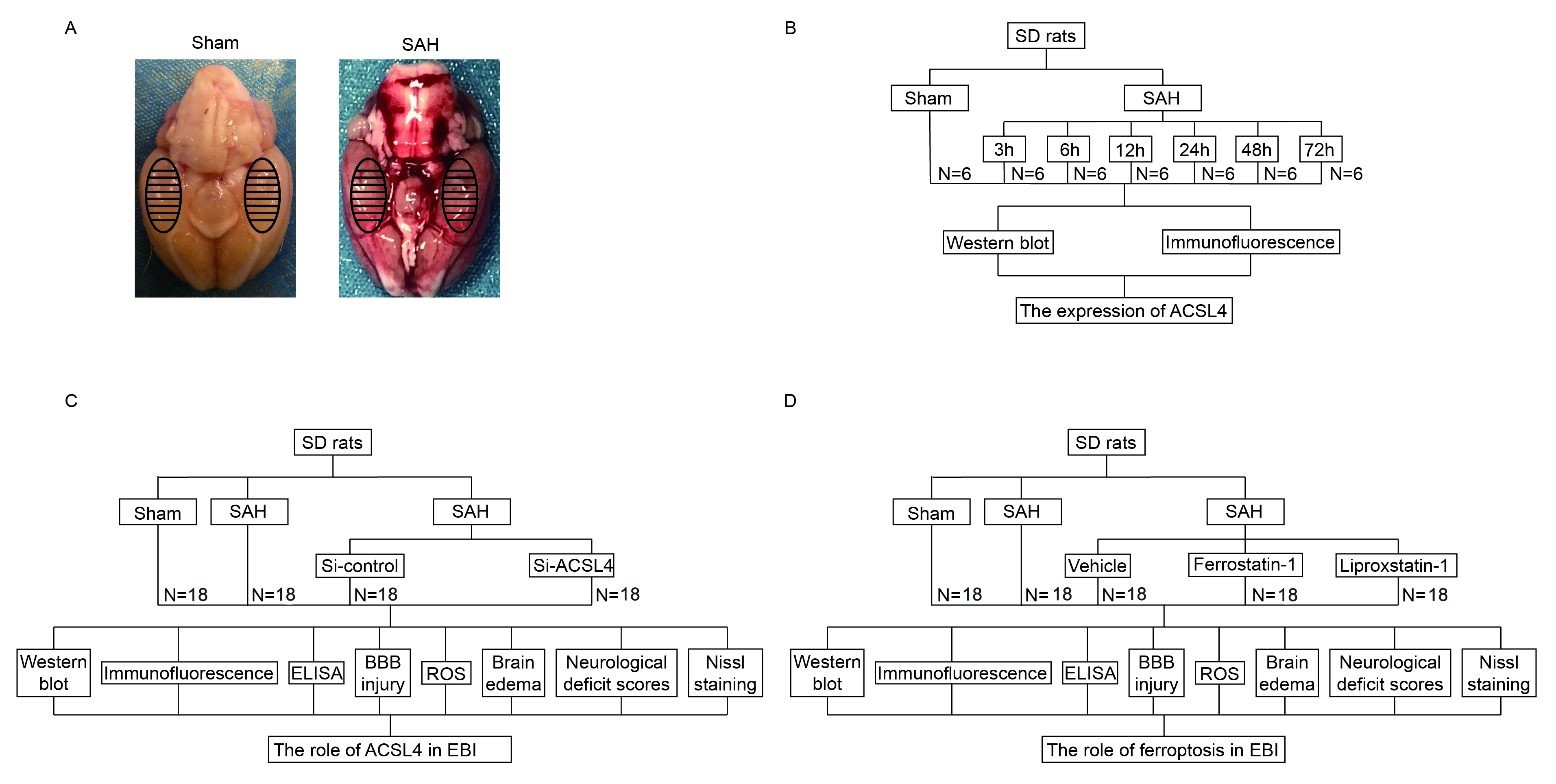

Supplement: Supplementary file 1 — Fig S1 [file CNS-27-449-s002.tif]

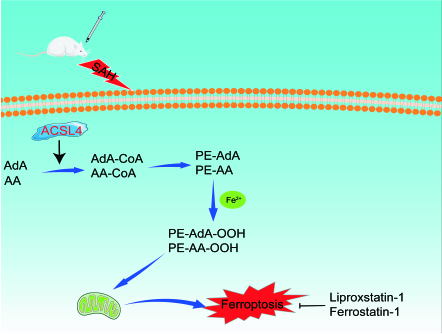

Supplement: Supplementary file 2 — Fig S2 [file CNS-27-449-s001.tif]
